# Interplay of Enzyme Therapy and Dietary Management of Murine Homocystinuria

**DOI:** 10.3390/nu12092895

**Published:** 2020-09-22

**Authors:** Insun Park, Erez M. Bublil, Frank Glavin, Tomas Majtan

**Affiliations:** 1 Section of Genetics and Metabolism, Department of Pediatrics, University of Colorado Anschutz Medical Campus, Aurora, CO 80045, USA; insun.park@ucdenver.edu; 2 Orphan Technologies Ltd., 8640 Rapperswil, Switzerland; erez.b@neopharmgroup.com (E.M.B.); frank.glavin@neovii.com (F.G.)

**Keywords:** inborn error of metabolism, rare disease, tandem mass spectrometry, mouse model, enzyme replacement therapy, sulfur metabolism, homocysteine

## Abstract

Albeit effective, methionine/protein restriction in the management of classical homocystinuria (HCU) is suboptimal and hard to follow. To address unmet need, we developed an enzyme therapy (OT-58), which effectively corrected disease symptoms in various mouse models of HCU in the absence of methionine restriction. Here we evaluated short- and long-term efficacy of OT-58 on the background of current dietary management of HCU. Methionine restriction resulted in the lowering of total homocysteine (tHcy) by 38–63% directly proportional to a decreased methionine intake (50–12.5% of normal). Supplemental betaine resulted in additional lowering of tHcy. OT-58 successfully competed with betaine and normalized tHcy on the background of reduced methionine intake, while substantially lowering tHcy in mice on normal methionine intake. Betaine was less effective in lowering tHcy on the background of normal or increased methionine intake, while exacerbating hypermethioninemia. OT-58 markedly reduced both hyperhomocysteinemia and hypermethioninemia caused by the diets and betaine in HCU mice. Withdrawal of betaine did not affect improved metabolic balance, which was established and solely maintained by OT-58 during periods of fluctuating dietary methionine intake. Taken together, OT-58 may represent novel, highly effective enzyme therapy for HCU performing optimally in the presence or absence of dietary management of HCU.

## 1. Introduction

**Figure 1:**
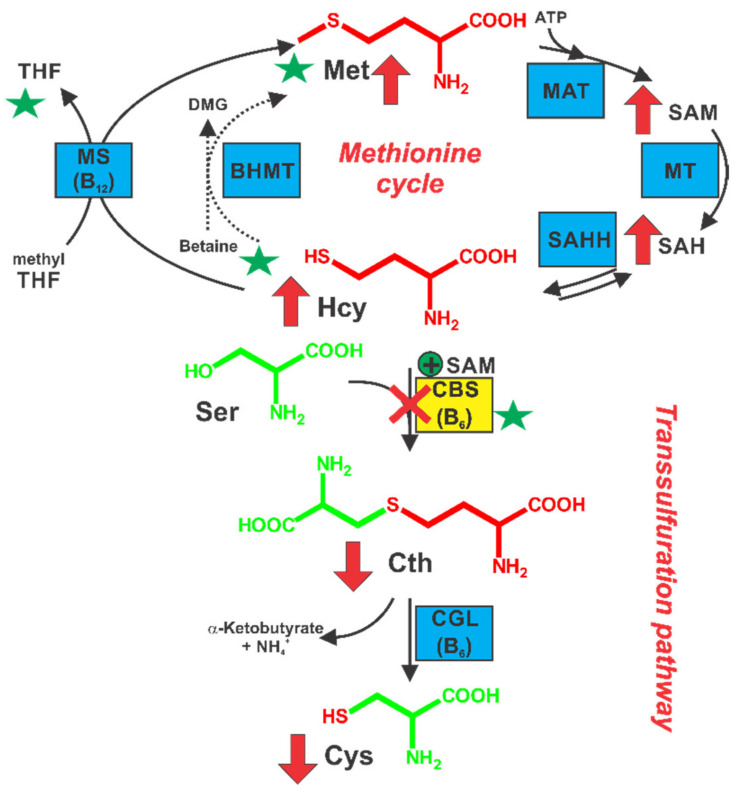
Sulfur amino acid metabolism and management of homocystinuria (HCU). In HCU, sulfur amino acid metabolism is disrupted due to deficient cystathionine beta-synthase (CBS) activity (red cross), which leads to the elevation of upstream metabolites homocysteine (Hcy), methionine (Met), S-adenosylmethionine (SAM), and S-adenosylhomocysteine (SAH) and decrease of downstream metabolites cystathionine (Cth) and cysteine (Cys) as indicated by red arrows. Green stars designate locations, where the current treatments for HCU work: restriction of dietary Met and protein intake to decrease production of Hcy, pyridoxine supplementation to stimulate residual CBS activity and folate/betaine supplementation to promote Hcy re-methylation back to Met. cystathionine γ-lyase (CGL); di-MethylGlyoxime (DMG); S-adenosylhomocysteine hydrolase (SAHH); Bet-Hcy-methyltransferase (BHMT).

Classical homocystinuria (HCU; OMIM# 236200) is a rare inborn error of sulfur amino acid metabolism caused by substantially reduced or entirely missing cystathionine beta-synthase (CBS) activity [1]. Human CBS is a complex enzyme with intricate regulation responsible for the condensation of homocysteine (Hcy), a toxic intermediate of methionine (Met) metabolism, with serine to form cystathionine (Cth), thus directing the flux of organic sulfur irreversibly toward the biosynthesis of cysteine (Cys) [2] (Figure 1). Consequently, a lack of CBS activity leads to extremely elevated plasma and tissue Hcy concentrations, which represent a biochemical hallmark of HCU. The clinical consequences include skeletal and connective tissue defects, impaired vision, mental retardation, and vascular complications, particularly stroke and deep vein thrombosis, which often lead to the premature death of HCU patients [1]. The major objective of treatment is to reduce the accumulation of Hcy and maintain it as close as possible to normal levels (5–15 µM; Figure 1). Recent guidelines for the management of HCU recommend maintaining plasma total Hcy (tHcy) below the 100 µM threshold, which, rather than being an ideal target for Hcy levels, represents a high but achievable target that may help in reducing some of the clinical complications [3]. Interestingly, a 50 µM tHcy threshold was recommended for the less affected individuals, so-called pyridoxine responders, in whom residual CBS activity can be stimulated by supplemental pyridoxine (vitamin B_6_). Its active form pyridoxal-5′-phosphate represents a catalytic cofactor of CBS. Different plasma tHcy thresholds for the two groups of patients affected by HCU and buildup of Hcy testify that these levels were set not as desired or ideal but rather as achievable targets that are attainable by the current standard therapy, thus further emphasizing unmet need. In addition, the majority of patients respond only partially to pyridoxine or not at all. The metabolic balance of HCU patients is thus controlled by dietary protein/Met restriction to decrease flux in the organic sulfur metabolism. In addition, betaine (Bet) supplementation was found beneficial to patients struggling with their diet to achieve therapeutic goals. Although dietary management could be quite effective, the poor taste of Met-free formula or Bet and the fishy odor resulting from Bet administration lead to poor compliance and subsequent exacerbation of clinical complications [1,3,4].

To address unmet need, we developed OT-58, a novel enzyme therapy for HCU [5,6,7,8]. OT-58 is a recombinant, truncated, human CBS C15S variant modified with 20 kDa linear N-hydroxysuccinimide ester-activated polyethylene glycol chains. When administered subcutaneously to various mouse models of HCU, OT-58 caused substantial reduction of tHcy and correction of metabolic balance. More importantly, the improved biochemical profile resulted in the correction of survival, liver disease [6], facial alopecia, ciliary zonules [7], endothelial dysfunction, cognitive impairment, body composition, and bone mineralization in HCU mice [8]. Notably, most of these results were accomplished in HCU mice maintained on unrestricted Met intake in the form of standard rodent chow with no additional supplements. However, for real-world scenarios of protein/Met restriction, Bet supplementation, and poor compliance with these dietary measures, it is important to understand how OT-58 performs under the challenge of current mainstay therapies, which reduce the availability of Hcy substrate (Met restriction) or directly compete for it (Bet supplementation). Therefore, we evaluated the short- and long-term efficacy of OT-58 to correct the metabolic profile of HCU mice on the background of graded Met restriction, Cys or Bet supplementation, withdrawal of Bet, and fluctuations in Met intake to model current dietary management strategies treating HCU and poor adherence of patients to the dietary management of HCU.

## 2. Materials and Methods

### 2.1. Test Compounds

OT-58, a human, truncated CBS carrying C15S mutation covalently modified with a linear 20 kDa N-hydroxysuccinimide ester polyethylene glycol (ME-200GS; NOF), was prepared as described elsewhere [5]. The OT-58 batch used in the study was 1618TR1 with a protein concentration of 22 mg/mL and average CBS specific activity of 1332 ± 45 U/mg of protein.

### 2.2. Diets

Prior to the study execution, animals were maintained on a standard complete extruded diet 2920X (Envigo). Amino acid-defined diets (Envigo) varying in content of sulfur amino acids Met or Cys were used in the below described animal studies. Their compositions and nutritional information are summarized in Table 1. Briefly, the diet designated MET4.0 containing 4.0 g/kg Met and 3.5 g/kg Cys (as cystine) had similar content of sulfur amino acids as the standard complete diet 2920X (5 g/kg Met and 3 g/kg Cys) and thus served as a normal control diet. Diets designated MET2.0, MET1.0, and MET 0.5 had reduced contents of Met compared to MET4.0 to 2.0, 1.0, and 0.5 g/kg, respectively. Similarly, the diet designated as MET8.2 had roughly doubled the Met content (8.2 g/kg) compared to MET4.0. The content of Cys in all the MET diets was identical (i.e., 3.5 g/kg). In contrast, the diet designated as 3xCYS had Met content same as the MET4.0, but its Cys content was tripled to 10.5 g/kg. When indicated, drinking water through lixit was replaced by a bottle containing drinking water with 2% betaine hydrochloride (Bet; Sigma).

### 2.3. Animals and Study Design

All procedures were performed at the University of Colorado Anschutz Medical Campus under the Institutional Animal Care and Use Committee (IACUC)-approved protocol# 81, which complied with the Guide for the Care and Use of Laboratory Animals. The University is an Association for Assessment and Accreditation of Laboratory Animal Care (AAALAC)-accredited (#00235), Public Health Service (PHS)-assured (#D16-00171), and the United States Department of Agriculture (USDA)-licensed (#84-R-0059) institution. “Human Only” (HO) mice were previously generated in our laboratory [9] and propagated and genotyped as described previously [10]. Both male and female young-adult HO mice (20–30 weeks of age) were used in two separate studies reported here. At the end of each study, mice were euthanized by CO_2_ asphyxiation followed by cervical dislocation.

**Figure 2:**
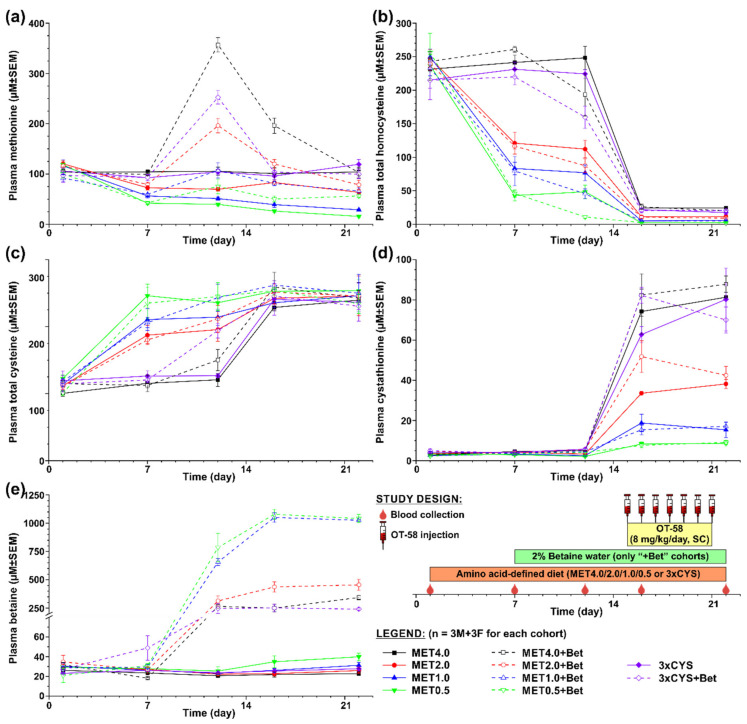
Short-term evaluation of OT-58 on the background of current standard of care for HCU. Ten HO mouse cohorts (n = 3 males + 3 females each) were set on a different dietary regime evaluating normal or decreased Met content in the diet (MET4.0/2.0/1.0/0.5), Bet supplementation (2% Bet water), and dietary Cys supplementation (3xCYS). After acclimation, the efficacy of OT-58 treatment was assessed according to the study design shown in the bottom right corner. (**a**) Plasma Met, (**b**) plasma tHcy, (**c**) plasma tCys, (**d**) plasma Cth, (**e**) plasma Bet. Solid lines and closed symbols represent cohorts without Bet supplementation, while dashed lines and open symbols denote cohorts with Bet supplementation. Data points and error bars represent means and standard errors of means, respectively.

The first study was designed to evaluate the efficacy of OT-58 treatment in the context of normal or reduced dietary Met intake without or with Bet or Cys supplementation. Figure 2 shows a graphical representation of the study design. Briefly, ten cohorts of HO mice (n = 3 males + 3 females each) were transferred from the 2920X diet to amino acid-defined MET4.0/2.0/1.0/0.5 or 3xCYS diets. After a week of acclimation to new diets, drinking water in half of the cohorts was replaced with 2% Bet water (“+Bet” cohorts). After another week, all cohorts received seven doses of OT-58 (8 mg/kg subcutaneously (SC)) once daily. Blood samples were collected on the following days: D1 (prior to the new diets), D7 (prior to the Bet supplementation), D12 (3 days before the first dose of OT-58), D16 and D22 (24 h after the first and last OT-58 dose, respectively).

**Figure 3:**
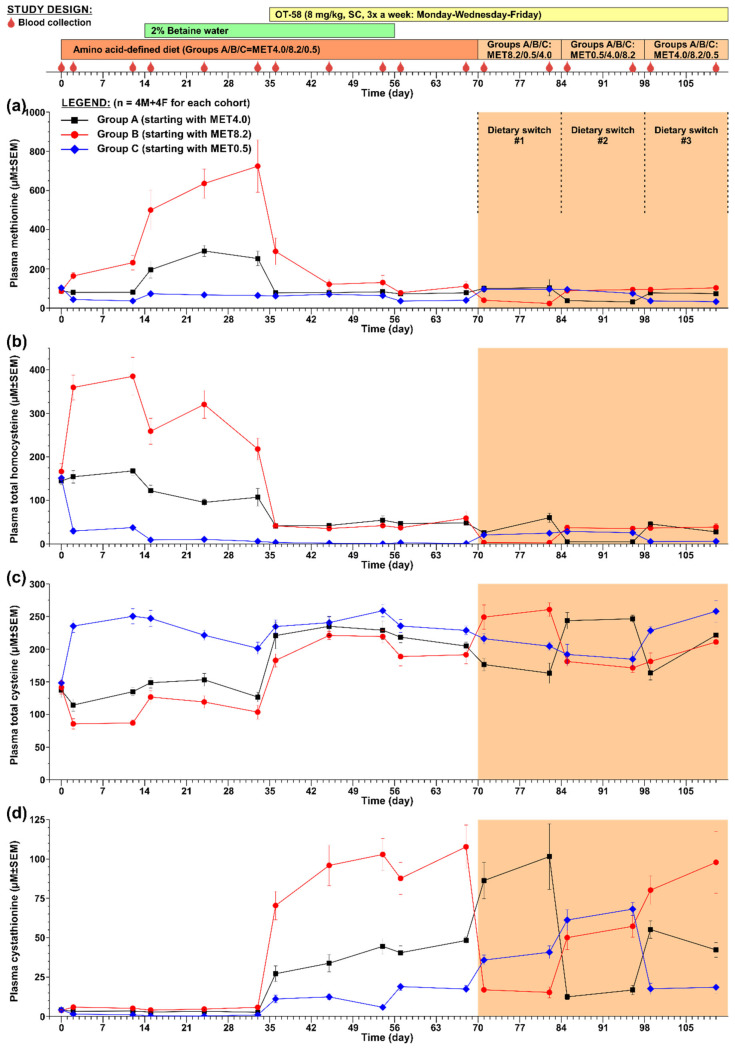
Long-term evaluation of OT-58. Three HO mouse cohorts (n = 4 males + 4 females each) were set on diets with reduced (MET0.5), normal (MET4.0), and increased (MET8.2) Met content later combined with Bet supplementation. OT-58 efficacy was evaluated on the background of these dietary regimes and later challenged by switching cohorts between the three diets as outlined in study design shown at the top. (**a**) Plasma Met, (**b**) plasma tHcy, (**c**) plasma tCys, (**d**) plasma Cth. Data points and error bars represent means and standard errors of means, respectively.

The second study was designed to evaluate the efficacy of OT-58 treatment in the context of reduced, normal, and increased Met intake without or with Bet supplementation followed by fluctuations in Met intake. Figure 3 shows a graphical representation of the study design. Briefly, three cohorts of HO mice (n = 4 males + 4 females each) were transferred from the 2920X diet to amino acid-defined MET4.0/8.2/0.5 diets. After two weeks of acclimation to new diets, drinking water for all cohorts was replaced with 2% Bet water. After three weeks of acclimation to the diets supplemented with Bet, all cohorts started to receive OT-58 (8 mg/kg SC) 3x a week (Monday–Wednesday–Friday) until the end of the study. After three weeks on diets, Bet supplementation and OT-58 injections, Bet was discontinued and replaced by regular drinking water through lixit. Two weeks after Bet withdrawal, fluctuations in Met intake were induced by switching between the three diets every two weeks as outlined in Figure 3. Blood samples were collected on the following days: D1 (prior to the new diets); D2 and D12 (acclimation to the new diets); D15, D24, and D33 (acclimation to Bet supplementation); D36, D45, and D54 (acclimation to OT-58 treatment); D57 and D68 (withdrawal of Bet); D71 and D82 (diet switch#1); D85 and D96 (diet switch#2); and lastly D99 and D110 (diet switch#3).

### 2.4. Blood Collection and Analysis

Single-use lancets for submandibular bleedings were used for blood collections into BD Microtainer tubes with lithium heparin (Becto-Dickinson). The tubes were then centrifuged at 10,000× *g* for 5 min, followed by the transfer of plasma into 1.5 mL tubes and storage at −80 °C. Sulfur amino acid metabolites, namely Met, tHcy, total cysteine (tCys), Cth, and Bet, were determined by stable-isotope-dilution liquid chromatography tandem mass spectrometry (LC–MS/MS) as described elsewhere [11].

### 2.5. Statistical Analysis

All data are presented as mean ± standard error of the mean (SEM). Statistical analyses were conducted by ANOVA followed by Tukey’s multiple comparison test to determine significance, with values of *p* < 0.05 being significant.

## 3. Results

### 3.1. Short-Term Evaluation of OT-58 on the Background of Current Standard of Care for HCU

In the first study, shown in Figure 2, we evaluated the impact of current standard of care for HCU, i.e., dietary Met restriction often combined with Bet and/or Cys supplementation, on the plasma metabolic profile of HO mice in the absence and presence of OT-58. Five different diets were tested varying in content of Met (MET4.0/2.0/1.0/0.5) and Cys (3xCYS), with MET4.0 representing standard sulfur amino acid intake, MET2.0/1.0/0.5 representing graded Met restriction (50/25/12.5% methionine content of MET4.0), and 3xCYS representing dietary supplementation of cysteine (3× more Cys compared to MET4.0 with the same Met content). Additionally, Bet supplementation was tested in conjunction with each of the diets (“+Bet” cohorts). Lastly, OT-58 (8 mg/kg/day, SC) was administered once daily for 7 days to all cohorts in order to study interaction of novel enzyme therapy with current standard of care in HO model of HCU.

Plasma Met decreased on average by 38%, 48%, and 63% from the initial levels (107 ± 3 µM on average) in mice fed with MET2.0, MET1.0, and MET0.5 diets, respectively (*p* < 0.05) but remained similar in mice on MET4.0 and 3xCYS (Figure 2a). Similarly, plasma tHcy declined on average by 52%, 67%, and 81% from the initial levels (237 ± 4 µM on average) in mice fed with MET2.0, MET1.0, and MET0.5 diets, respectively (*p* < 0.01) but remained unchanged in mice on MET4.0 and 3xCYS (Figure 2b). Consequently, plasma tCys increased on average to 168%, 182%, and 208% of the initial levels (113 ± 2 µM on average) in mice on MET2.0, MET1.0, and MET0.5 diets, respectively (*p* < 0.05) but remained stable on MET4.0 and 3xCYS (Figure 2c). Plasma Cth and Bet remained largely unchanged during the acclimation period of mice to new diets (3.7 ± 0.3 and 28.3 ± 1.9 µM on average, respectively; Figure 2d,e).

Bet supplementation resulted in a massive increase of plasma Bet from the initial levels on average 10-fold in mice on the MET4.0, MET2.0, and 3xCYS diets and 26-fold in mice on the MET1.0 and MET0.5 diets (*p* < 0.01; Figure 2e). Elevated Bet resulted in plasma tHcy reduction by ~26% from the initial levels in mice on the MET4.0 and 3xCYS diets (*p* < 0.05) and further reduced tHcy from the concentrations achieved by reduced Met intake by 25%, 43%, and 76% in mice on the MET2.0, MET1.0, and MET0.5 diets, respectively (*p* < 0.05; Figure 2b). Consequently, plasma Met increased by Bet supplementation in correlation with Met intake and the initial plasma Met concentrations by 1.8-, 1.8-, and 2.5-times in mice on the MET0.5, MET1.0, and MET2.0, respectively, and 2.7- and 3.6-times in mice on the 3xCYS and MET4.0, respectively (*p* < 0.05; (Figure 2a). Bet supplementation also improved plasma tCys by ~18% in mice on the MET2.0 and MET1.0 compared to that in those on diet alone and by 34% and 61% in mice on the MET4.0 and 3xCYS diets, respectively (*p* < 0.05; Figure 2c). Plasma Cth remained unchanged with Bet supplementation (Figure 2d).

Treatment with OT-58 normalized plasma tHcy in mice on the MET2.0/1.0/0.5 with or without Bet supplementation to 10, 5, and 2 µM, respectively, and substantially reduced it to 25 and 20 µM in mice on the MET4.0 and 3xCYS with or without Bet supplementation, respectively (*p* < 0.01; Figure 2b). Plasma tCys was also normalized in all cohorts on average to 245 µM representing 2.2-fold increase compared to the initial levels (*p* < 0.05; Figure 2c). As anticipated, OT-58 resulted in marked accumulation of plasma Cth, which correlated with Met content in the diets but was not significantly affected by Bet supplementation. Specifically, plasma Cth increased compared to the initial levels on average 27-, 16-, 10-, 5-, and 3-fold in mice on MET4.0, 3xCYS, MET2.0, MET1.0, and MET0.5, respectively (*p* < 0.01; Figure 2d). Following OT-58 treatment, plasma Met decreased to levels similar to those induced by the diets alone. However, OT-58 resulted in an additional plasma Met drop by 40% and 50% in mice the MET1.0 and MET0.5, respectively, compared to pre-OT-58 period (*p* < 0.05; Figure 2a), which was completely prevented by Bet supplementation. Plasma Bet remained unchanged with OT-58 treatment (Figure 2e).

### 3.2. Long-Term Evaluation of OT-58

In the second study, shown in Figure 3, we evaluated the efficacy of OT-58 treatment to improve the plasma metabolic balance of HO mice in the context of reduced (MET0.5), normal (MET4.0), and increased (MET8.2) Met intake with or without Bet supplementation followed by fluctuations in Met intake to model for periods of non-compliance of HCU patients with the dietary regime.

Acclimation of HO mice to new diets resulted in a change of plasma Met concentrations in correlation with dietary Met intake. Specifically, plasma Met in mice on the MET4.0 remained unchanged, while it dropped by 65% in those on the MET0.5 and increased by 270% in those on the MET8.2 compared to the initial concentrations (*p* < 0.01; Figure 3a). Plasma tHcy followed a similar trend as that of Met, i.e., remained similar on the MET4.0, decreased by 75% on the MET0.5, and increased by 232% on the MET8.2 compared to the initial concentrations (*p* < 0.01; Figure 3b). Conversely, plasma tCys levels were essentially normalized by the MET0.5 (~243 µM; *p* < 0.01), remained unchanged on the MET4.0, and decreased by 38% on the MET8.2 compared to the initial levels (*p* < 0.05; Figure 3c).

Bet supplementation exacerbated hypermethioninemia in HO mice on the MET8.2 additionally by up to 3.1-fold compared to the diet alone and increased plasma Met 3.1- and 1.7-fold in HO mice on the MET4.0 and MET0.5, respectively (*p* < 0.05; Figure 3a). However, plasma tHcy was reduced by 36%, 43%, and 85% compared to the diet alone in mice on the MET4.0, MET8.2, and MET0.5, respectively (*p* < 0.05; Figure 3b), with its concentration normalized in the cohort on the MET0.5 (~8 µM). Bet supplementation did not significantly change plasma tCys in HO mice on the MET4.0 and MET0.5 but improved it by ~20% in those on the MET8.2 (*p* < 0.05; Figure 3c).

Treatment with OT-58 further decreased tHcy in mice on the MET0.5 diet to ~2 µM and substantially reduced hyperhomocysteinemia in HO mice on the MET4.0/8.2 to ~43 µM (*p* < 0.01; Figure 3b). Plasma Met remained unchanged in mice on the MET0.5 but was substantially decreased in those on the MET4.0 and MET8.2 to ~80 and ~125 µM, respectively (*p* < 0.05; Figure 3a). On the other hand, plasma tCys increased and essentially normalized in all cohorts (MET0.5/4.0/8.2 = ~250/234/220 µM, *p* < 0.05; Figure 3c). Catalytic action of OT-58 resulted in marked accumulation of plasma Cth, which correlated with Met content in the diets. Specifically, plasma Cth increased compared to the initial levels on average 2.4-/8.6-/23.1-fold in mice on the MET0.5/4.0/8.2, respectively (*p* < 0.01; Figure 3d).

Withdrawal of Bet supplementation did not affect plasma tHcy levels in either cohort (Figure 3b). Plasma Met decreased on average by 41%, 10%, and 27% in mice on the MET0.5/4.0/8.2, respectively (*p* < 0.05; Figure 3a). Plasma tCys also decreased slightly by on average 10%, 8%, and 13% in mice on the MET0.5/4.0/8.2, respectively (*p* < 0.05; Figure 3c) but remained within or close to normal levels (232/211/190 µM vs. 233 µM in historical heterozygous HO controls [12]). Plasma Cth did not dramatically change after Bet withdrawal except in mice on the MET0.5, where it increased by ~3.1-fold compared to the value prior to the Bet withdrawal (*p* < 0.01; Figure 3d).

In order to model for periods of non-compliance of HCU patients with their Met-restricted dietary regime, fluctuations of Met intake were achieved by three diet switch periods, each lasting for two weeks. Thus, each cohort received the MET0.5, MET4.0, and MET8.2 for two weeks and ended on the same diet as before the diet switch period (see Figure 3 for study design). The results could be summarized as follows. Plasma tHcy was maintained in the 20–60 µM range except for periods on the MET0.5, when it was normalized and fluctuated around 5 µM (Figure 3b). Plasma tCys remained high and close to normal with the MET0.5 diet always contributing to full normalization (Figure 3c). Plasma Met remained stable and similar to the initial levels but always decreased on average by 63% when switched to the MET0.5 (Figure 3a). Lastly, plasma Cth remained markedly elevated compared to the initial levels due to OT-58 action on Hcy and correlated with the Met intake during each specific diet-switch period. Higher Met intake in the MET8.2 led to the highest plasma Cth concentrations (up to 101 µM), while lower Met intake in the MET0.5 led to the lowest plasma Cth concentrations (up to 18 µM; Figure 3d).

## 4. Discussion

Dietary management currently represents a primary therapeutic approach to pyridoxine non-responsive CBS-deficient HCU and an additional option for partially responsive HCU patients [3]. The first case report of successful dietary management of HCU with a diet low in Met and supplemented with cystine was published in 1966 by Komrower et al. [13] just a few years after HCU was first described in 1963 by Carson et al. [14]. Such dietary intervention resulted in a decreased influx of sulfur and, thus, lower production and accumulation of Hcy in affected individuals. As an elevated concentration of plasma Hcy has become a well-known risk factor for thromboembolic complications in HCU patients, Bet supplementation of either Met-restricted or unrestricted diet was introduced in early 1980s and showed further reduction or complete normalization of plasma Hcy levels [15,16]. This improvement, however, came at the price of elevation of plasma Met. Multiple studies have shown that loss of metabolic control is associated with serious complications, and, thus, HCU treatment must continue throughout the entire life [1,3,4,17]. However, compliance with Met restriction or Bet supplementation often deteriorates, particularly during adolescence, or is poor in general in individuals diagnosed later in life [4].

Due to the rarity of classical HCU and lack of appropriate animal models for many years, there has not been any development of new treatments for HCU until recently. To address unmet need and to provide new therapeutic options, we developed OT-58 and successfully evaluated it using various mouse models of HCU that are now available [5,6,7]. OT-58 performed well, correcting the plasma metabolic profile of HCU mice maintained on protein/Met unrestricted standard chow. In real-world scenarios, however, OT-58 efficacy will be confronted by the mainstay therapy (Met restriction, Cys and Bet supplementations) and lapses in compliance with the mainstay therapy (e.g., variable Met/protein intake, Bet withdrawal). Therefore, to gain further insight into the interplay of dietary management of HCU with OT-58, here we evaluated the short- and long-term efficacy of OT-58 on the background of increased, normal, and reduced Met intake with or without Cys or Bet supplementation. The effect of OT-58 treatment on both the biochemical markers and the symptoms of the disease in mice was extensively studied in our lab using multiple mouse models of HCU [6,7,8]. The effect on biomarkers was similar in all mouse models. As it takes a longer time to see the impact of treatment on symptoms of HCU than the periods of various treatments included in this study, we decided to use HO mouse model. The HO mice recapitulate biochemical imbalance of HCU very well and do not suffer from poor survival or other adverse manifestations compared to other models available.

Met-restricted diets were effective in lowering of both plasma Met and tHcy, which decreased proportionally to the degree of Met restriction (Figure 2). However, even the most restrictive diet containing 12.5% of Met compared to control amino acid-defined MET4.0 diet (or 10% compared to standard complete diet) did not achieve normalization. MET0.5 decreased initial tHcy from 237 and 151 µM to 47 and 34 µM, respectively (Figure 2 and Figure 3). In an I278T transgenic mouse model of HCU characterized by more severe phenotype and higher basal tHcy, severe Met restriction (i.e., 8.5–12.5% Met compared to standard diet) reduced tHcy from 357 and 423 µM to 81 and 140 µM, respectively [8,18]. Together, the data suggest that the capacity of severe Met restriction to reduced tHcy depends on severity of the disease, level of CBS impairment, and, thus, initial tHcy concentration. Importantly, OT-58 normalized tHcy when combined with some level of Met restriction (MET2.0/1.0/0.5) and substantially reduced it when paired with normal or increased Met intake (MET4.0/8.2; Figure 2 and Figure 3). These results indicate high efficacy of OT-58 to decrease and maintain tHcy below the recommended threshold of 100 µM to prevent serious clinical complication in HCU patients [3]. Interestingly, a combination of OT-58 with MET1.0 and MET0.5 resulted in continuous decline of plasma Met (Figure 2). Low plasma Met is likely beneficial for HCU, as well as other indications, such as various cancers [19], but in case of HCU, the level of Met restriction should still allow for normal growth and development [3]. Our data suggest that OT-58 in combination with moderate or no Met restriction markedly reduced plasma tHcy while it sustained normal plasma Met, which in turn should allow for normal growth and development. Accordingly, OT-58 may potentially offer a significant diet relaxation opportunity for human patients.

Cys supplementation on the background of normal Met intake failed to improve both plasma tHcy and tCys (Figure 2). This was unexpected, as previous study on I278T mice showed that 40 mM N-acetyl-L-cysteine, a more stable and water soluble Cys precursor, in drinking water increased tCys by 50% and reduced tHcy by 13% compared to levels in untreated I278T mice [20]. Either Cys supplementation (12.4 mM in drinking water) or taurine (160 mM in drinking water) normalized hepatic and blood taurine levels in HO mice, which lead to the normalization of HCU-induced regulatory changes in expression of the enzymes of Cys catabolic pathways [21]. However, no other improvements in phenotype of HCU mice were observed suggesting that low tCys may not be directly related to major clinical symptoms of HCU, thus, putting Cys supplementation in question. In fact, as the relationship between Cys and Hcy as thiol redox-active compounds is complex [22], data suggest that lowering of tHcy is more effective strategy to achieve tCys normalization compared to Cys supplementation. However, further research is clearly needed to clarify the role of Cys supplementation in HCU. The efficacy of OT-58 did not seem to be affected by 3-fold dietary Cys excess on the background of normal Met intake (Figure 2).

Bet supplementation should be considered as an adjunctive treatment in less compliant HCU patients who fail to achieve target levels of tHcy [3,23]. The use of Bet is generally safe, although its unpleasant taste and fishy odor often result in poor compliance [4]. In addition, extreme elevation of plasma Met during Bet treatment has been found to cause cerebral edemas, which resolve after Bet discontinuation [24,25]. Indeed, Bet supplementation resulted in plasma Met increase directly proportional to Met intake, while partially lowering tHcy (Figure 2 and Figure 3). Interestingly, there was a marked variability in plasma betaine concentrations among mouse cohorts on different Met intake (Figure 2). While cohorts on Met4.0/2.0 showed 250–450 µM Bet, mice on the lower Met intake (MET1.0/0.5 diets) achieved plasma Bet concentrations of 650–1050 µM. We hypothesize that the difference is related to intracellular availability of Hcy substrate for Bet-Hcy-methyltransferase (BHMT) and to the level of BHMT expression. Indeed, increased Met intake resulted in higher production of Hcy, which was available for BHMT-catalyzed re-methylation with Bet back to Met resulting in lower plasma Bet concentrations and increased plasma Met concentrations (Figure 2a,e). In addition, plasma tCys increased with Bet supplementation (Figure 2c). This effect can simply be explained by the fact that Hcy binds to Cys residues of plasma proteins (as well as free Cys) with a higher affinity than that of Cys thus replacing Cys, which results in plasma tCys decrease in HCU. Consequently, any treatment or intervention that reduces plasma tHcy prevents the loss of Cys and leads to the correction of plasma tCys. The efficacy of Bet in lowering tHcy diminished significantly over time in HO mice [26], which was later found to be alleviated by taurine treatment, which induced Bet-repressed expression of BHMT [27]. However, the long-term efficacy of Bet in HCU patients does not seem to be compromised [28]. Our studies showed that OT-58 provided additional benefit in correcting metabolic balance in HO mice over Bet supplementation alone and successfully competed with Bet for Hcy substrate. Regardless of Met restriction, OT-58 alone lowered tHcy to the same levels as achieved by co-administered Bet and OT-58 (Figure 2). Furthermore, OT-58 alone maintained plasma tHcy and improved metabolic control after Bet withdrawal irrespective of dietary Met intake (Figure 3). On the other hand, Bet supplementation maintained plasma Met on the background of severe Met restriction (12.5–25% of normal intake), while co-administered OT-58 further decreased plasma Met in HO mice on MET1.0/0.5 diets (Figure 2). It is important to note the OT-58 does not replace the defective intracellular CBS. Whereas the native enzyme functions inside the cells, OT-58 does not enter the cells and operates in bloodstream, thus lowers intracellular Hcy indirectly. Betaine, on that other hand, serves as a methyl donor in the cytoplasm, and its therapeutic potential depends on the activity of intracellular BHMT to re-methylate Hcy to Met (Figure 1). Accordingly, OT-58 does not directly compete with betaine for Hcy inside the cells. In addition, Bet promotes the conservation of sulfur in the Met cycle. OT-58, on the other hand, processes Hcy irreversibly to Cth and, thus, removes the sulfur from the Met cycle (Figure 1). Together, our data suggest that Bet supplementation without severe Met restriction does not offer additional benefits when co-administered with OT-58. Moreover, exposure of HCU mice to OT-58 in the absence of Bet and without being primed with Bet supplementation was extensively studied by our group [6,7,8], showing Bet supplementation is not required to achieve good metabolic control on a background of normal Met intake combined with OT-58 treatment.

As OT-58 is currently tested in a phase I/II clinical trial (ClinicalTrials.gov ID NCT03406611), better understanding of OT-58 efficacy and its interaction with the current therapeutic options for HCU is important and highly relevant for study design, evaluation, and interpretation of the clinical trial results. Our previous data and those presented in this study clearly show that OT-58 retains its efficacy and performs well in a range of Met intakes in mouse models of HCU with different residual CBS activities [12]. Similarly, it is expected that these encouraging pre-clinical data may be translated to HCU patients, and, thus, OT-58 may become a valuable tool in the management and treatment of HCU. Specifically, OT-58 can efficiently reduce plasma tHcy below the 100 µM threshold or normalize it entirely (Figure 2 and Figure 3). Co-administration of Bet or Cys or their absence did not significantly affect OT-58′s efficacy in improving metabolic balance (Figure 2). OT-58 maintained improved metabolic balance during periods of highly variable dietary Met intake (Figure 2 and Figure 3). Lastly, our previous long-term studies using different mouse models of HCU showed no reduction of OT-58 efficacy over time, indicating no significant immune response to OT-58 in mice [6,7,8], although the possibility that OT-58 will induce neutralizing antibodies in some HCU patients cannot be ruled out. Taken together, this pre-clinical study on HO mouse model of HCU suggests that OT-58 may represent a novel, highly effective, enzyme therapy for HCU performing optimally in the presence or absence of previous treatments, such as Met/protein restriction or Cys/Bet supplementation and during periods of poor compliance of HCU patients with dietary management of the disease. It remains to be seen how OT-58 will perform in the real world, but our study gives an optimistic outlook for this and similar enzyme therapies in development for HCU [29].

## Figures and Tables

**Table 1:** Amino acid-defined diets used in the study.

Component (g/kg)	MET4.0 (TD.170063)	MET2.0 (TD.170062)	MET1.0 (TD.170061)	MET0.5 (TD.110591)	3xCYS (TD.170065)	MET8.2 (TD.01084)
L-Alanine	3.5	3.5	3.5	3.5	3.5	3.5
L-Arginine.HCl	12.1	12.1	12.1	12.1	12.1	12.1
L-Asparagine	6.0	6.0	6.0	6.0	6.0	6.0
L-Aspartic Acid	3.5	3.5	3.5	3.5	3.5	3.5
L-Cystine	3.5	3.5	3.5	3.5	10.5	3.5
L-Glutamic Acid	36.5	38.5	39.5	40.0	29.5	40.0
Glycine	23.04	23.04	23.04	23.04	23.04	23.3
L-Histidine.HClxH_2_O	4.5	4.5	4.5	4.5	4.5	4.5
L-Isoleucine	8.2	8.2	8.2	8.2	8.2	8.2
L-Leucine	11.1	11.1	11.1	11.1	11.1	11.1
L-Lysine.HCl	18.0	18.0	18.0	18.0	18.0	18.0
L-Methionine	4.0	2.0	1.0	0.5	4.0	8.2
L-Phynylalanine	7.5	7.5	7.5	7.5	7.5	7.5
L-Proline	3.5	3.5	3.5	3.5	3.5	3.5
L-Serine	3.5	3.5	3.5	3.5	3.5	3.5
L-Threonine	8.2	8.2	8.2	8.2	8.2	8.2
L-Tryptophan	1.8	1.8	1.8	1.8	1.8	1.8
L-Tyrosine	5.0	5.0	5.0	5.0	5.0	5.0
L-Valine	8.2	8.2	8.2	8.2	8.2	8.2
Sucrose	353.14	353.14	353.14	353.14	353.14	344.98
Corn Starch	150.0	150.0	150.0	150.0	150.0	150.0
Maltodextrin	150.0	150.0	150.0	150.0	150.0	150.0
Soybean Oil	80.0	80.0	80.0	80.0	80.0	80.0
Cellulose	30.0	30.0	30.0	30.0	30.0	30.0
Mineral Mix (AIN-93M-MX)	35.0	35.0	35.0	35.0	35.0	35.0
Ca(H_2_PO_4_)2xH_2_O	8.2	8.2	8.2	8.2	8.2	8.2
Vitamin Mix (AIN-93-VX)	19.5	19.5	19.5	19.5	19.5	19.5
Choline bitartarate	2.5	2.5	2.5	2.5	2.5	2.7
TBHQ (antioxidant)	0.02	0.02	0.02	0.02	0.02	0.02
**Summary Nutrient Information** (**% by weight/% kcal from**)						
Proteins	14.9/15.1	14.9/15.1	14.9/15.1	16.5/16.4	15.0/15.2	17.3/17.2
Carbohydrates	65.7/66.7	65.7/66.7	65.7/66.6	65.7/65.6	65.7/66.6	64.9/64.8
Fat	8.0/18.3	8.0/18.3	8.0/18.3	8.0/18.0	8.0/18.3	8.0/18.0
Energy density (Kcal/g)	3.9	3.9	3.9	4.0	3.9	4.0

Met: Methionine.
